# Compromised Regeneration, Damage to Blood Vessels and the Endomysium Underpin Permanent Muscle Damage Induced by Puff Adder (*Bitis arietans*) Venom

**DOI:** 10.3390/toxins17100496

**Published:** 2025-10-06

**Authors:** Sodiq Opeyemi Adeyemi, Nicholas John Richards, Ali Alqallaf, Husain Bin Haidar, Mustafa Jawad Jalil Al-Asady, Jarred Williams, José R. Almeida, Ketan Patel

**Affiliations:** 1 School of Biological Sciences, University of Reading, Reading RG6 6UB, UK; 2 Medical Services Authority, Ministry of Defence, Kuwait City 13012, Kuwait; 3 Ministry of Higher Education and Scientific Research, Baghdad 8CH9+3JH, Iraq; 4 School of Pharmacy, University of Reading, Reading RG6 6UB, UKj.r.dealmeida@reading.ac.uk (J.R.A.); 5 Biomolecules Discovery Group, Universidad Regional Amazónica Ikiam, Tena 150150, Ecuador

**Keywords:** Puff adder, *Bitis arietans*, muscle, regeneration, necrosis, fibrosis

## Abstract

The puff adder (*Bitis arietans*) is a clinically relevant viper species found throughout Africa, and it is responsible for a greater incidence of health-related envenomations than all other snake species on the continent combined. Unresolved skeletal muscle damage is a common consequence of *B. arietans* envenomation that can result in long-term morbidity and even death. Antivenom treatment can mitigate the systemic effects of the venom but offers little protection against local tissue damage. Identifying the mechanisms through which *B. arietans* venom induces tissue damage and impedes skeletal muscle regeneration could identify possible treatment alternatives that could help alleviate the long-term consequences of envenomation. Skeletal muscle has an innate ability to regenerate, but constituents within the venom can impede multiple stages of this regeneration process. In this study, we employed a combination of biochemical analyses, cell-based assays, and in vivo experiments to assess the toxicological implications of *B. arietans* envenomation and its impacts on key processes of regeneration. Our findings demonstrate that the pathological characteristics of permanent muscle damage resulting from *B. arietans* envenomation may be attributed to the venom’s effects on muscle stem cell precursors, the extracellular matrix (ECM), and the influence of blood-borne proteins that promote fibrosis.

## 1. Introduction

Snakebite envenomation affects countless lives and communities worldwide, especially in developing tropical countries [1]. It is classified as a neglected tropical disease by the World Heath Organization, with roughly 4.5–5.4 million people falling victim to snakebites annually, resulting in approximately 1.8–2.7 million cases of envenomation and 81,000–138,000 mortalities [1,2]. Snakebite envenomation leads to about 270,000–300,000 cases and 32,000–38,000 deaths annually in Africa alone [3,4,5], with *B. arietans* being responsible for more envenomation than all other snake species on the continent combined [6]. In Nigeria, the incidence of injury caused by *B. arietans* is estimated to be 15,000–20,000, with 2000 deaths annually [7]. *B. arietans* is recognised as the most medically significant viper species on the African continent, contributing to the highest rates of mortality and morbidity associated with snakebite envenomation [7,8]. The venom of *B. arietans* is highly potent, resulting in severe tissue damage, bleeding abnormalities, and, in some cases, organ failure [9,10,11]. A single viper bite can inject a substantial volume of venom, significantly elevating the risk of fatality or serious health issues if not treated promptly [12]. The cryptic camouflage and ambush predation strategies of *B. arietans* make it challenging to detect, as it often remains motionless. This behaviour increases the likelihood of accidental encounters with humans and animals, leading to defensive strikes [13]. The high frequency of venomous bites can be attributed to *B. arietans*’ extensive distribution and frequent interactions with humans.

Skeletal muscle is often the primary affected tissue by *B. arietans* envenomation and there can often be long-term consequences for the victim if they survive. However, this is not the case for all snakes. Some venoms, especially from Elapids, that contain high levels of phospholipaseA_2_ (PLA_2_), induce systemic myotoxicity [14,15]. This results in myofibre death, causing clinical features that manifest as rhabdomyolysis. Elevated levels of myoglobin and creatine kinase in the blood are key signs of this condition, which can lead to acute kidney injury [16]. However, as these venoms seem to spare the basal lamina of the muscle fibres as well as capillaries and neuromuscular junctions, the innate regeneration mechanisms are able to fully restore tissue integrity [17]. In contrast, venoms from viper species not only have some PLAs activity but high levels of other more injurious molecules that affect muscle components other than only muscle fibres [18].

Skeletal muscle possesses an exceptional capacity for regeneration, which is primarily mediated by resident stem cells known as satellite cells [17,19]. The regeneration of skeletal muscle after damage is a highly coordinated process that involves not only these stem cells but also numerous other critical players, both cellular and acellular [20]. Under optimal conditions, muscle tissue can regenerate from a state of complete damage to full recovery within a few weeks [21,22]. Recent studies have identified key components of muscle regeneration that may be disrupted in various skeletal muscle diseases and in response to snake envenomation [23,24,25,26,27]. During the regeneration process, muscle damage is rapidly followed by the infiltration of neutrophils and the activation of monocytes. These cells secrete factors that create an inflammatory environment, which serves two purposes: first, to clear away the debris from dead myofibres, and second, to stimulate satellite cells to transition from a quiescent state to an active one that promotes proliferation and migration to sites of tissue damage [28,29,30,31,32,33]. This is followed by a pro-resolution phase, in which a subset of macrophages promotes muscle cell differentiation [34].

A significant question that arises is why, despite the inherent regenerative capacity of skeletal muscle, it does not exhibit the same regenerative response following envenomation by *B. arietans*. Identifying the phases of innate regeneration that are disrupted constitutes the first step in developing treatments for this debilitating condition. Here, we used a combination of biochemistry, cell-based assays, and in vivo studies to show that the pathological features of permanent muscle damage caused by *B. arietans* envenomation could be due to the action of the venom on muscle stem cell precursors, the extracellular matrix, and the effect of blood-borne proteins that promote fibrosis.

## 2. Results

**Figure 1:**
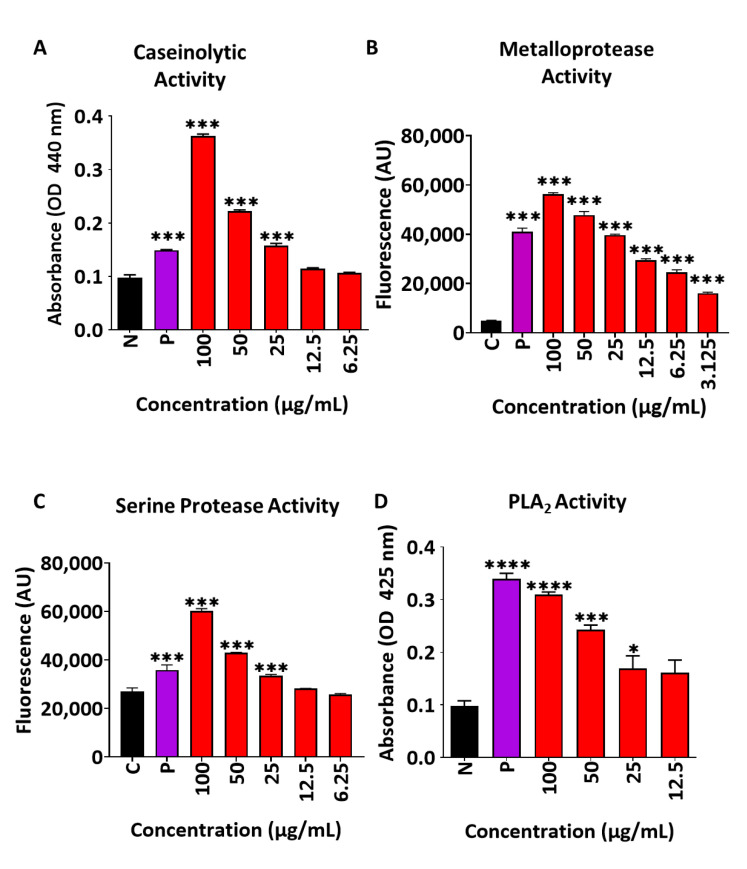
Profiling enzyme activity of *B. arietans* venom. (**A**) Caseinolytic activity quantification using azocasein as substrate. *B. arietans* venom used at 6.25 to 100 μg/mL. *C. atrox* venom (50 μg/mL) used as positive control. (**B**) Metalloprotease activity profiling using DQ-gelatine. *B. arietans* venom used at 3.125 to 100 μg/mL. *C. atrox* venom (50 μg/mL) used as positive control. (**C**) Serine protease activity quantification using BAAMC as a substrate. *B. arietans* venom used at 6.25 to 100 μg/mL. *C. atrox* venom (50 μg/mL) used as positive control. (**D**) Phospholipase A_2_ activity measurements in *B. arietans* venom (used at 12.5 to 100 μg/mL) using diheptanoyl thio-PC as substrate. *D. russelii* venom (50 μg/mL) used as positive control (*n* = 3). Data shown with ± SEM, *p*-values were calculated by one-way ANOVA followed by the Bonferroni post hoc test (* = *p* < 0.05, *** = *p* < 0.001,**** = *p* < 0.0001).

The investigation began by profiling the general enzymatic activity in the *B. arietans* venom used in this entire study. We first turned to the caseinolytic assay, which detects the liberation of tyrosine from casein due to the proteolytic activity of the venom. Using this assay, we found that the *B. arietans* venom had robust proteolytic properties and had higher activity than the control sample (*Crolatus atrox*) when used at equal concentrations (Figure 1A). A number of studies have described the components of *B. arietans* venom and hence we investigate it in more detail to identify the specific types of molecules that might be responsible for the general proteolytic activity described in Figure 1A. We first turn to quantify the levels of metalloprotease activity using the DQ-gelatine assay. Profiling of this activity showed that there was considerable metalloprotease activity in *B. arietans* venom and that it was greater in comparison to the *C. atrox* sample at equal concentrations (Figure 1B). *B. arietans* venom at 42.85 μg/mL had 50% metalloprotease activity. Next, we profiled serine protease activity, to find high levels of activity for this enzyme in *B. arietans* venom: greater than an equal concentration to the venom from *C. atrox* (Figure 1C), with 50% activity of 49.86 μg/mL. Finally, profiling levels of PLA_2_ showed that there was activity for this subclass of protein. However, it was lower than equal concentrations of the positive control (which in this case was venom from *Daboia russelii*-Russell’s viper, Figure 1D), with 50% activity at 30.0 μg/mL. Hence, the *B. arietans* venom used in this study shows presence of metalloprotease, serine protease activity, and PLA_2_ activity.

**Figure 2:**
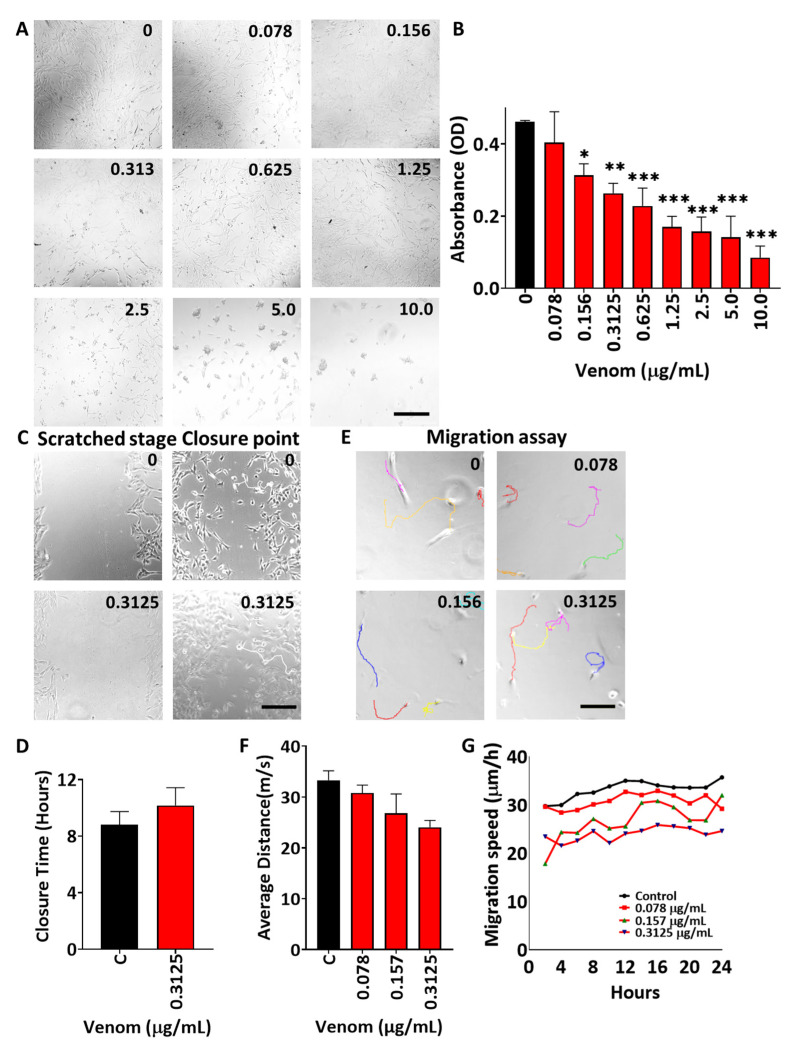
Effect of *B. arietans* venom on muscle cell viability and migration. (**A**) C2C12 cell images after 24 h exposure to venom at different concentrations (μg/mL) in all images. (**B**) Quantification of cell viability using the MTS assay at various venom concentrations (concentration of venom used is in black in top right corner in all images). (**C**) Scratch assay cell at start and end of experiment with time of closure in red. (**D**) Quantification of scratch closure. (**E**) Migration tracks of individual C2C12 cells (coloured lines) followed for a period of 24 h in various concentrations of venom. (**F**) Quantification of distance travelled by individual cells at different concentrations of venom over 24 h. (**G**) Profile of C2C12 speed of individual cell over a 24 h period. For B and D, data represents the mean ± SEM (n = 3). For F and G, data represent the mean ± SEM (n = 16). The scale bars represent 200 µm in all the images. The *p*-value shown (* *p* < 0.05, ** *p* < 0.01, and *** *p* < 0.001) was calculated using either Student’s *t*-test or one-way ANOVA followed by the Bonferroni post hoc test.

Next, we explored the impact of *B. arietans* venom using in vitro assays reporting on the early stages of muscle regeneration. The study began with the profiling of the impact of the venom on cell proliferation and survival using the mouse C2C12 line. The MTS assay that we used is a proxy for cell number. Herein, we found that a very low concentration (0.078 μg/mL) had no effect on cell number, but all higher concentrations impacted the MTS readout (Figure 2A,B). Importantly for this study was the observation that at concentrations of 0.313 μg/mL and lower, cells appeared normal in the presence of venom for at least 24 h. (Figure 2A). At concentrations of 0.625 and 1.25 μg/mL, the vast number of cells still appeared similar to controls, although there was sporadic presence of rounded entities (Figure 2A). Venom concentrations above 1.25 μg/mL resulted in the development of abnormal cell morphologies, most likely due to the induction of cell death. Based on these findings, further in vitro experiments were conducted using venom at 0.3125 μg/mL or lower.

Thereafter, we used the data from the proliferation/viability assessment to determine the impact of *B. arietans* venom at sublethal concentrations on other aspects of early muscle development. The impact of *B. arietans* venom on cell migration was assessed using two different approaches, using either the scratch assay protocol, which investigates bulk cell movement, or time-lapse profiling of isolated individual cells. We found that *B. arietans* venom failed to impact migration (Figure 2C–G).

**Figure 3:**
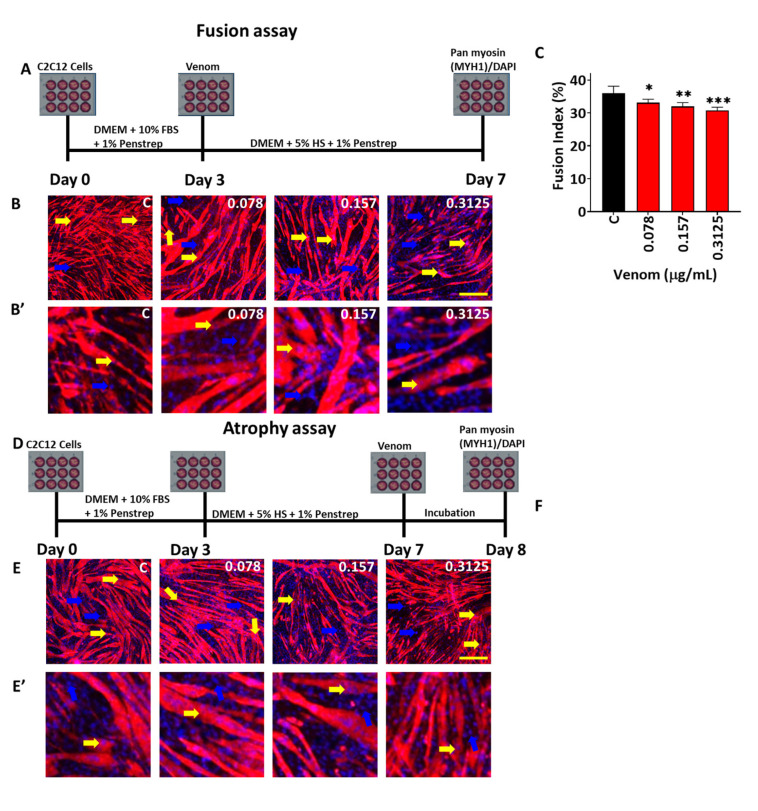
*B. arietans* venom inhibits myoblast fusion and induces myotubes atrophy. (**A**) Schematic representation of the protocol used for fusion assay. (**B**) Staining of myotubes for pan myosin-heavy chain (MYHC) (yellow arrow) and DAPI (blue arrows) on day 7 (yellow arrows showing MYHC-expressing cells with nuclei; blue arrows, cell nuclei not in MYHC-expressing cells). (**B’**) High magnifications of B with same labelling. (**C**) Quantification of the fusion index of the myotubes (n = 12). (**D**) Schematic representation of protocol used for atrophy assay. (**E**) Staining of myotubes (yellow arrow showing MYHC-expressing cells containing nuclei; blue arrows, cell nuclei not in MYHC-expressing cells). (**E’**) High magnifications of E with same labelling. (**F**) Quantification of the myotube area (n = 20). Data represent the mean ± SEM. *p* values shown were calculated by one-way ANOVA followed by the Bonferroni post hoc test (* *p* < 0.05, ** *p* < 0.001 and *** *p* < 0.001) using GraphPad Prism 10. The scale bars represent 200 µm.

Next, we examined the impact of *B. arietans* venom on myoblast fusion. Herein, C2C12 cells were propagated in growth media until they reached 80% confluency before exchanging media to one that supports differentiation. At the same time, we introduced the *B. arietans* venom. Following a period of 96 h, the cells were fixed and processed for immunocytochemistry to detect myosin-heavy chains. The fusion index was then calculated, which revealed that all used venom concentrations decreased this metric (Figure 3A–C).

Lastly, we examined the impact of *B. arietans* venom on myotube atrophy. Here, myotubes were developed (4 days in differentiation media) before the addition of venom for a period of 24 h. The size of differentiated myotubes (expressing pan MYHC) was measured to reveal that all concentrations of venom caused a decrease in this metric compared to controls (Figure 3D–F). Therefore, of the four early stages of muscle development, we found that *B. arietans* venom affected survival, fusion, and myotube atrophy, but not migration.

**Figure 4:**
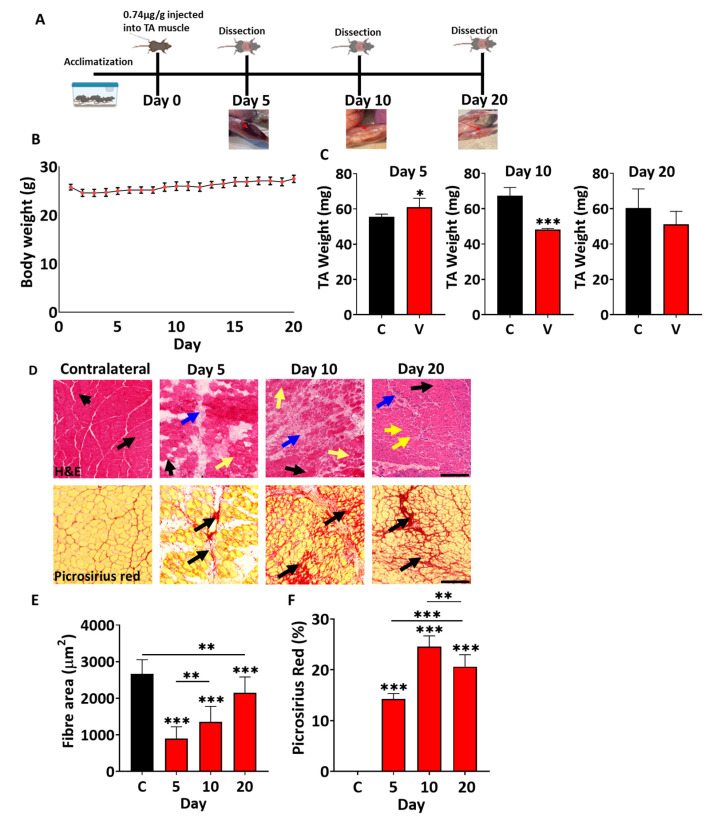
*B. arietans* venom affects tibialis anterior (TA) muscle weight and structure in mice. (**A**) Graphic of the protocol used for the in vivo muscle damage experiment. Images show damaged muscle collected at different times. Red arrows indicate damage to muscle. (**B**) Body weight of the venom-injected C57BL/6 mice. (**C**) Weight of venom-injected TA muscles of C57BL/6 mice compared to contralateral muscles at different time points. (**D**) Top panel H and E: staining of the muscle sections (black arrow indicates fibre with peripheral nuclei, yellow arrows indicate the centrally located nuclei (CLN) in the regenerating fibres, and blue arrows shows infiltrating cells). Bottom panel Picrosirius red: staining of the muscle sections (black arrows indicate fibrosis). (**E**) Quantification of centrally located muscle fibre area (red bars). Black bar represents area of normal fibre (undamaged muscle fibre). (**F**) Fibrosis quantification of the area of regenerative fibres in selected 200 µm^2^ areas. Data represent the mean ± SEM (n = 5 mice in each cohort). *p* values (* *p* < 0.05, ** *p* < 0.01 and *** *p* < 0.001) shown were calculated by either Student’s *t*-test or one-way ANOVA followed by the Bonferroni post hoc test. The scale bars represent 200 µm.

We next examined the impact of *B. arietans* venom on mature skeletal muscle. To that end, it was injected at a dose of 0.74 µg/g body weight (determined through pilot study that induced Tibialis anterior (TA) muscle necrosis without effects on other muscles and animal health) into the TA muscle of adult mice. Thereafter, the TA muscle was collected at different times and processed for macroscopic and microscopic examination (Figure 4A). Monitoring of the mice following injection of venom into the TA muscle failed to reveal any general health issues.

We found that 5 days after venom injection, the TA muscle showed extensive signs of haemorrhaging, which resolved over time (Figure 4A). Although the body weight of mice did not change during the course of the study (Figure 4B), there was an initial increase in the mass of the damaged TAs at day 5, followed by a statistical drop in this metric at day 10, and thereafter an increase to reach a mass that was not significantly less than the un-injured contralateral muscle (Figure 4C). Hence from a macroscopic perspective, *B. arietans* venom induced changes in muscle that were partially resolved over time.

We then examined the muscle using histological staining. Inspection of tissue architecture with Haematoxylin and Eosin revealed extensive damage at day 5, with the destruction of myofibres after venom injection (Figure 4D). Thereafter, there was recovery, indicated by the development of regenerating muscle fibres and a decrease in infiltrating cells, which was still not complete by day 20 (Figure 4D). Quantification of regenerating fibres showed a gradual increase in size over time (Figure 4E). However, even at day 20, there were many infiltrating cells in between muscle fibres, and importantly, there was a considerable variation in muscle fibre size (Figure 4D). We next profiled the degree of fibrosis by examining muscle sections with Picrosirius red histology. We found extensive Picrosirius red staining as early as day 5 post venom injection, which increased to cover almost 25% of damaged regions by day 10, and then decreased at day 20 (Figure 4D,F). Hence, *B. arietans* venom induced significant muscle damage as well as damage to supporting cells (blood vessels, hence the early haematoma), which was only partially resolved by day 20. Even at this late time point, there was evidence of abnormal tissue development evidenced by the presence of extensive Picrosirius red staining.

**Figure 5:**
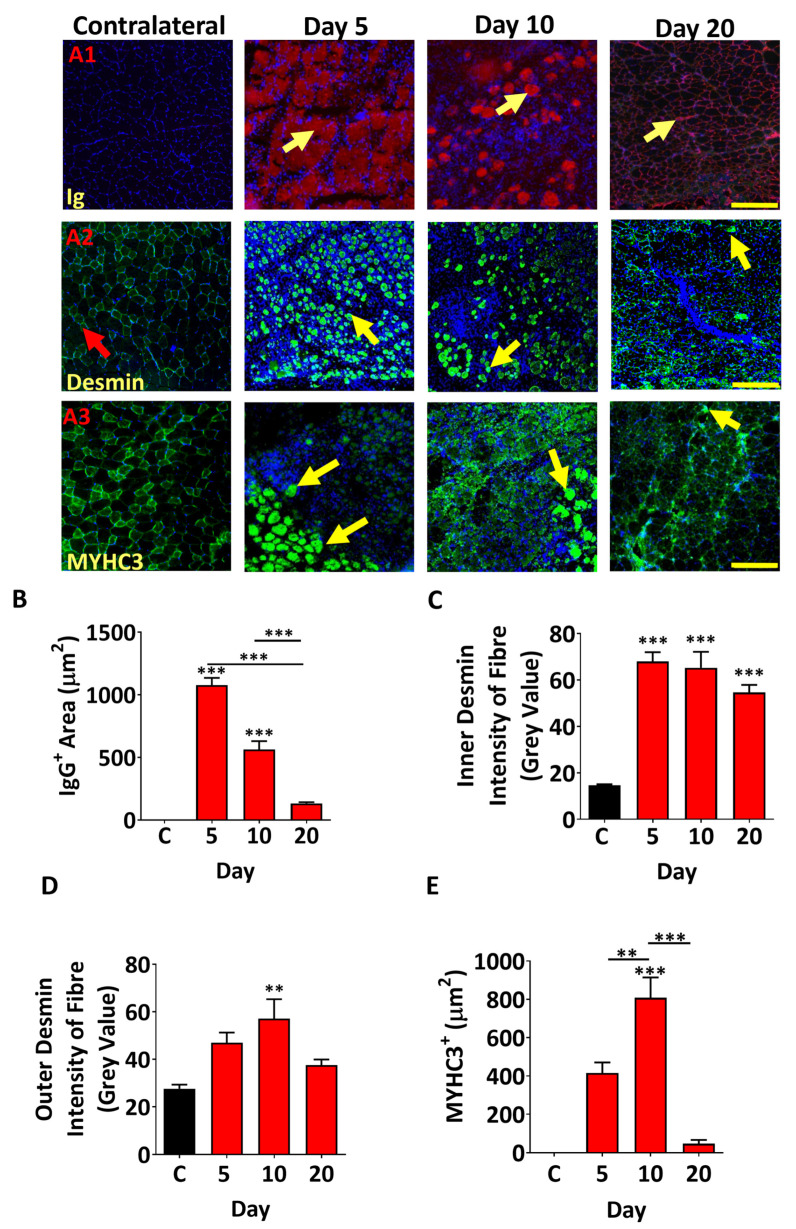
Profiling of muscle regeneration markers after damage induced by *B. arietans* venom in tibialis anterior TA muscle. (**A**) A1: top panel shows intra-fibre Ig localisation (yellow arrows). A2: expression of Desmin in regenerating muscle fibres. Expression of Desmin was located at fibre periphery in undamaged fibre (red arrows), whereas damaged fibres show expression in cytoplasm of cells/fibres (yellow arrows). A3: MYHC3 in regenerating muscle fibres (yellow arrows). (**B**) Quantification of infiltrated fibres. (**C**) Quantification of Desmin expression (intensity) inner part of myofibre. (**D**) Quantification of Desmin expression (intensity) at myofibre periphery. (**E**) Quantification of the MYHC3 expression domain in centrally located nuclei-containing myofibres. Comparisons made only between treated groups. Data represent the mean ± SEM (n = five mice in each cohort). *p* values (** *p* < 0.01 and *** *p* < 0.001) shown were calculated by one-way ANOVA followed by the Bonferroni post hoc test. The scale bar represents 200 µm images.

We next focused on the impact of the venom on muscle fibres and the process of regeneration. One of the earliest features of muscle fibre damage is the infiltration of circulating antibodies into fibres. We identified muscle fibres that contained mouse Ig molecules and found that at day 5, large fibres showed the presence of circulating proteins (Figure 5(A1)). Thereafter, the size of these fibres decreased (Figure 5(A1),B). Next, we examined the regeneration process by following the localisation of Desmin, which in mature fibres is predominantly found at the periphery. However, it is expressed throughout differentiating myoblasts and in immature fibres before becoming concentrated at the periphery with maturation [35,36].Our profiling of Desmin revealed the presence of cells expressing this protein throughout the cytoplasm at day 5 (Figure 5(A2)) and at very high levels at the centre and periphery (Figure 5C,D). The cytoplasmic localisation of Desmin was evident at day 10, whereas by day 20 it was generally decreased, but importantly, its normal localisation at the edge of the fibre was not evident (Figure 5(A2),C,D). Lastly, we examined the expression of embryonic Myosin Heavy Chain (MYHC3), which is used as a means to identify regenerating muscle fibres. We found small MYHC3-postive fibres at day 5, which increased in size by day 10 and were almost absent by day 20 (Figure 5(A3),E).

**Figure 6:**
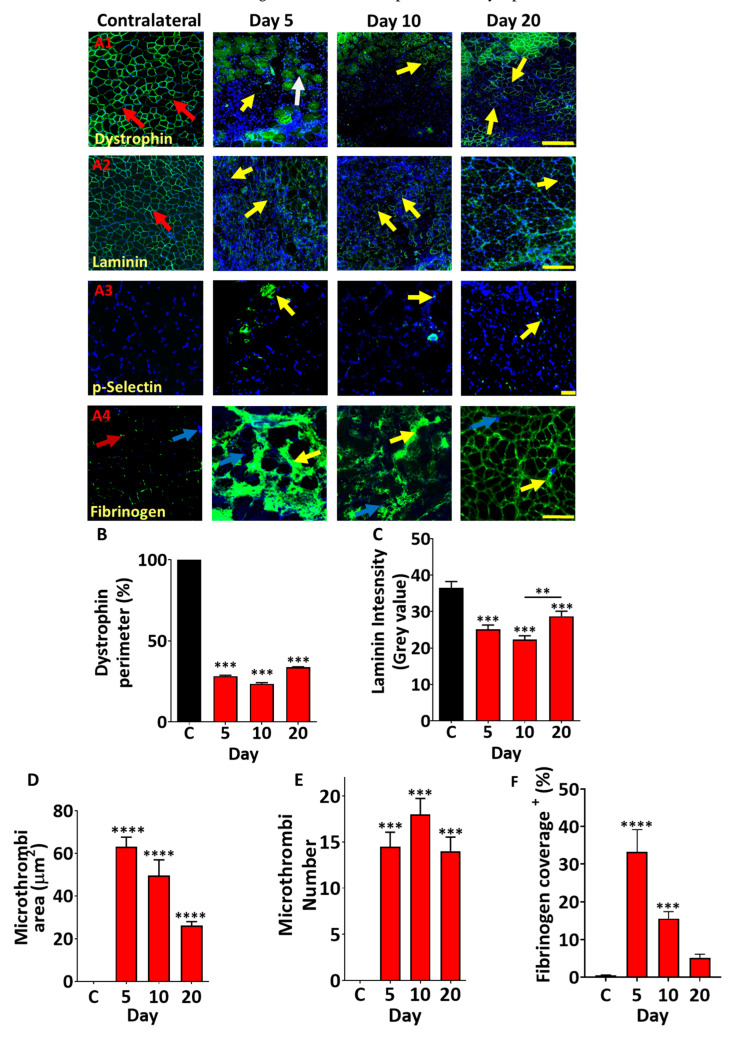
Profiling of ECM-associated proteins, platelet markers, and fibrinogen deposition following muscle damage induced by *B. arietans* venom. (**A**) A1: Dystrophin expression showing continuous expression or high circularity around undamaged fibres (red arrows). In damaged fibres, it is patchy or low circularity (yellow arrows) or in muscle fibres (white arrows). A2: Expression of Laminin showing continuous expression around undamaged fibres (red arrows). In damaged fibres it is patchy (yellow arrow). A3: pSelectin localisation in damaged regions (yellow arrows). A4: Fibrinogen deposition in damaged fibres (yellow arrows) with DAPI marking nuclei (blue arrows). (**B**) Quantification of dystrophin in damaged region. (**C**) Quantification of Laminin expression at myofibre periphery. (**D**) Quantification of microthrombi area in damaged region. (**E**) Quantification of microthrombi number. (**F**) Quantification of the fibrinogen as percentage area cover in damaged region. Data represent the mean ± SEM (n = five mice in each cohort). *p* values (** *p* < 0.01, *** *p* < 0.001 and **** *p* < 0.0001) shown were calculated by one-way ANOVA followed by the Bonferroni post hoc test. The scale bar represents 200 µm for A1 and A2 and 50 µm for A3.

Next, we investigated another important aspect of muscle regeneration, namely the extracellular matrix (ECM) and associated proteins. One of the key molecules that links the cytoskeleton of muscle fibres to the ECM and thereby imbues resistance to contraction-induced muscle damage is the protein Dystrophin. It is normally localised immediately inside the sarcolemma (Figure 6(A1)). We found that at an early stage of *B. arietans* venom-induced muscle damage, the expression of this vital protein was faint or discontinuous, and this was measured as circularity. Instead of being at the periphery of the fibre, we often found Dystrophin inside the fibres (Figure 6(A1)). Thereafter, the expression intensity decreased with very low circularity around the damaged fibres (at day 10) and then recovered partially by day 20 (Figure 6(A1)–B). However, even at day 20, there were still areas where there was no or only patchy expression of Dystrophin within individual muscle fibres (Figure 6(A1)). Quantification of Dystrophin at the sarcolemma revealed that at all stages examined, it was present at significantly lower levels than in control undamaged muscle (Figure 6B). Next, we examined the localisation of Laminin, one of the key ECM components of skeletal muscle. Similarly to Dystrophin, the expression of this protein was disturbed from its regular pattern 5 days after venom injection and thereafter decreased by day 10 to then increase by day 20, nevertheless failing to come back to normal levels at the end of the experimental period (Figure 6(A2),C). Next, having seen large depositions of blood at early stages post damage, we profiled the distribution of p-Selectin, a molecule found at high levels in platelets. In undamaged muscle, there was very weak, if any, signal for this molecule, which contrasted the situation at day 5 post damage, where large and numerous depositions were visible (Figure 6(A3),D,E). As seen in the macroscopic examination, the level of blood proteins thereafter cleared. However, even at day 20, there were signs of abnormal deposits of this platelet-derived protein. Lastly, we profiled the presence of molecules that facilitate interactions with Fibrinogen since binding in tissue is a near-universal sign of tissue injury or inflammation. We found extensive evidence for Fibrinogen in muscle 5 days post damage, which gradually decreased over time (Figure 6(A4),F). These results imply sustained leakiness of blood vessels following introduction of *B. arietans* venom.

## 3. Discussion

Long-term muscle/tissue damage is a common consequence of Puff adder (*B. arietans)* envenomation, resulting in long-term morbidities that affect the quality of life for bite victims and their families [2,37]. Bites from *B. arietans* are typically to the foot/leg and hands; the cytotoxic effects of the venom can cause severe tissue destruction, with characteristically poor regeneration. This damage and poor regeneration can manifest in loss of function or complete loss of limbs, rendering victims unable to work. Antivenom is the only currently approved treatment for snakebite envenomation, but its efficacy in mitigating local tissue damage is poor and often out of reach for many. The rural agricultural communities most affected by snakebite envenomation are typically characterised as having poor infrastructure, connectivity, and healthcare provision. This can delay victims from reaching healthcare professionals who have the resources and training to administer antivenom [38]. The combination of poor efficacy and availability does nothing to help mitigate the long-term morbidities associated with *B. arietans* envenomation. New treatment options need to be explored.

To start to develop new treatments, first, the mechanisms through which the venom induces muscle damage and impedes regeneration must be established [24]. Long-term muscle damage is only typically seen following viper envenomation; elapid envenomation is associated with lower incidences of severe long-term tissue damage [17]. This is a result of different venom compositions and the abundance of enzymatic cytotoxins. Viper venoms typically have high levels of snake venom metalloproteases, serine proteases and phospholipase A_2S_. Elapid venoms are dominated by low molecular weight non-enzymatic three-finger toxins and PLA_2S_ [39]. These components of elapid venoms can induce significant amounts of tissue damage, but muscle typically regenerates well following elapid envenomation [23]. Nevertheless, PLA_2S_ from either elapids or vipers are capable of inducing myofibre necrosis, which can be fully repaired [40]. PLA_2S_ seem to spare blood vessels, neuromuscular junctions, and the basal lamina of muscle fibre [19]. Snake Venom Metalloproteases (SVMPs) are regarded as the main mitigators of long-term muscle damage following viper envenomation as they affect a wide variety of structures in skeletal muscle [41]. With this in mind, we profiled and assessed the enzymatic activity of *B. arietans* venom, finding very high levels of metalloprotease and serine protease activity, as well as significant levels of PLA_2_ activity.

To assess the functional effects of this enzymatic activity, we employed in vitro and in vivo techniques, to elucidate the key mechanisms through which it induces tissue damage and impedes regeneration. C2C12_S_ are an immortalised mouse skeletal muscle precursor cell line that can be utilised to investigate the effects of venoms on cell viability and different processes of regeneration. MTS assays revealed that low concentration of *B. arietans* venom decreased proliferation, whereas high concentrations were toxic and caused cell death. Following these observations, an effective but sub-lethal dose was selected for investigation into its impacts on regenerative processes. The venom failed to impede the cells’ ability to migrate. However, it reduced myoblast fusion and the size of pre-formed myotubes. This highlights that *B. arietans* venom impedes many, but not all, key processes associated with early phases of muscle regeneration.

Our in vivo work showed that damage to a mouse TA muscle at a dose that affected only skeletal muscle was not fully resolved after 20 days. We found evidence that some features leading to good muscle regeneration were present, e.g., clearance of leaky muscle fibres. However, during this time, abnormal features including the development of fibrosis had also developed, which points to more long-term consequences of the insult. The results from this study offer a mechanistic insight into key elements disturbed by *B. arietans* venom.

We found that even after 10 days post injury, expression of Desmin was in the cytoplasm in a large number of tiny cells in the damaged region. This contrasts with its site of expression in muscle that has been damaged using cardiotoxin, which only affects mature muscle fibres; here it is found in its normal sub-sarcolemma position [35]. Indeed, the distribution of Desmin we reported here is highly reminiscent of its positioning in genetic models that specifically prevent myoblast fusion, such as the Myomaker null mutant [35]. Additionally, our in vitro work may explain the regenerated muscle fibres being smaller than those that were undamaged. Meyer (2018), amongst others, has shown that fibres regenerate to normal size in a matter of 14 days after CTX-induced damage [42]. In contrast, we showed that the regenerating fibres were smaller than in unaffected regions, even after 21 days. One possible explanation for this finding is our in vitro data set demonstrating that *B. arietans* venom can induce atrophy. For this line of reasoning to be feasible, it would necessitate that the venom is active at least until myotube fibres have formed, which takes a minimum of 3 days [35]. In support of this notion, we have shown that the venom components from another viper (*C. atrox*) are present following intramuscular injection for over a week [23]. This finding is in keeping with outcomes of other investigations of viper venoms. Saravia-Otten et al. (2013) reported that *B. asper* venom induced extensive damage when injected into the gastrocnemius muscle of mice [43]. Although the levels of venom were reduced greatly a few days after its introduction, homogenates from muscles collected at these time points still had significant levels of molecules that impaired myoblast proliferation and myotube formation [43,44]. Hence, we suggest that muscle regeneration could be impeded by *B. arietans* venom through its action on the survival of satellite cells progeny, their ability to fuse, and their capacity to induce atrophy of newly formed fibres.

Our work also sheds light on other mechanisms critical for muscle regeneration that could be disturbed by *B. arietans* venom. A large body of evidence has demonstrated that a continuous endomysium is required to support robust muscle regeneration (reviewed by Ahmad, et al. [45]). Details of the role played by the ECM during regeneration have recently by elegantly described by Collins et al. [29], who demonstrated that it acts as a dynamic jacket that firstly shrinks as muscle fibre proteins are cleared, and then acts to increase myoblast density to promote fusion [29]. This model holds that optimal muscle regeneration requires an intact ECM. However, we clearly show that this is not the case following the action of *B. arietans* venom; laminin expression was disrupted. Hence, according to the model of Collins et al. [29], muscle regeneration would be impeded since the critical density of myoblasts needed for new fibre formation would not be reached in the shorter period. This mechanism would also be hindered by our finding that *B. arietans* venom limits myoblast fusion.

Finally, our findings based on profiling blood-borne factors may explain the development of fibrosis. We showed that *B. arietans* venom induced significant damage to the circulatory system, evidenced by the presence of considerable amounts of blood-based proteins in damaged muscle at day 20. This is likely to be caused by the action of SVMPs, which are abundant in viper venoms that act through their ability to attenuate the barrier function of capillaries, as well as by attacking smooth muscle cells [46,47]. The outcome of these events could impede muscle regeneration at many levels. First and foremost, many studies have shown that a functioning circulatory system is a key determinant of effective muscle regeneration enabling the delivery of key cells and molecules to the damaged area [20]. Secondly, it is known that the initial phase of muscle damage is accompanied by inflammation. However, a compromised circulatory system could lead to victims’ own tissues producing molecules that are also injurious, a collection of molecules that Harris and Cullen call ‘autocoids’ [48]. Additionally, we suggest that platelets could have a role to play in the development of fibrosis following muscle damage induced by *B. arietans* venom. Herein, we suggest that damage to the blood vessels (supported by the presence of p-Selectin and Fibrinogen in muscle fibres) allows factors in blood to deregulate the action of fibroblasts and macrophages that ultimately induce the deposition of fibrotic tissue. This line of thinking is supported by work carried out in the context of neuromuscular disorders that have demonstrated the ability of fibrinogen to either directly stimulate collagen production by fibroblasts or reach this end point indirectly by first inducing the expression of TGF-β production by macrophages [49]. There is ample evidence that fibrinogen induces fibrosis not only in skeletal muscle but many other organs, including the kidney. Hence, we propose that targeting the action of blood-borne factors, including Fibrinogen, could be a means of attenuating fibrosis following venom-induced muscle damage, as well as in other pathologies.

## 4. Recommendations

In summary, our work further supports the notion put forward by us and others [17] that in order to develop a potent regime to attenuate the impact of viper envenomation the following need to be achieved: (1) protecting muscle cells, (2) preventing ECM damage, (3) preventing damage to blood vessels, and (4) addressing uncontrolled inflammation.

## 5. Materials and Methods

### 5.1. Preparation of Venom Aliquots

Lyophilised *B. arietans* venom was obtained from Venomtech Ltd., Kent, United Kingdom. Lyophilised *C. atrox* and *Daboia russelii* venoms were purchased (Sigma Aldrich, Poole, UK and LATOXAN, Portes-lès-Valence, France) and subsequently stored at −80 °C. A stock solution of 2 mg/mL was prepared in phosphate-buffered saline (PBS) and stored as aliquots at −80 °C until needed to minimise the risk of component degradation from freeze–thaw cycles.

### 5.2. Biochemical Activity

The enzymatic assays for the venom were performed as described previously by other studies [50,51,52]. Briefly, to assess general protease activity, chromogenic azocasein (Merck, Gillingham, UK) was used as a substrate at a concentration of 5 mg/mL in a 50 mM Tris-HCl buffer (pH 8.0). Venom at different concentrations were diluted in 90 µL of the substrate solution. After incubating for 90 min at 37 °C, 200 µL of 5% trichloroacetic acid (TCA) was added to each reaction and centrifuged for 5 min at 8000 rpm. The supernatant (150 µL) was placed in each well of a 96-well plate, to which 150 µL of NaOH (0.5 M) was added. The formation of TCA-soluble azopeptides was measured at 440 nm using a spectrofluorometer (FLUOstar OPTIMA, BMG Labtech, Ortenberg, Germany).

A dye-quenched (DQ) gelatine (ThermoFisher Scientific, Loughborough, UK) (50 µg/mL) was used as a fluorescent substrate for metalloprotease activity. The substrate was incubated with venom at various concentrations in 100 µL total reaction volumes at 37 °C, and fluorescence readings were taken every 10 min for 90 min with a spectrofluorometer (FLUOstar OPTIMA, BMG Labtech, Germany) at an excitation wavelength of 485 nm and emission at 520 nm. *C. atrox* venom was used as a positive control.

To measure venom serine protease activity, the substrate NαBenzoyl-L-Arginine-7-Amido-4-methylcoumarin hydrochloride (BAAMC) (2 µM) was incubated with various venom concentrations for 90 min, with fluorescence readings taken every 10 min on a spectrofluorometer (FLUOstar OPTIMA, BMG Labtech, Germany), using an excitation wavelength of 366 nm and emission at 460 nm. *C. atrox* venom (50 μg/mL) was used as a positive control.

PLA_2_ activity of *B. arietans* venom was assessed by evaluating its ability to hydrolyse a non-micellar synthetic substrate, 4-nitro-3-octanoyloxy benzoic acid (NOB), using a protocol optimised for 96-well plates [51]. Briefly, in a clear 96-well plate, 20 μL of NOB (3.1 mg/mL; Abcam (Cambridge, UK), ab141769) dissolved in acetonitrile was mixed with 20 μL of H_2_O and 200 μL of PLA_2_ reaction buffer (10 mM Tris-HCl, 10 mM CaCl_2_, 100 mM NaCl). Subsequently, 20 μL of varying concentrations of *B. arietans* venom were added to initiate the reaction. Absorbance at 425 nm was recorded immediately using a FLUOstar Omega (BMG LABTECH) plate reader over the course of one hour, and the final absorbance values were used for comparisons. *D. russelii venom* (50 μg/mL) was used as a positive control.

### 5.3. Cell Culture

The immortalised mouse myoblast C2C12 line (ATCC, USA) was used for this study [51]. The C2C12 cells were cultured in “growth media” [high glucose DMEM with 10% (*v*/*v*) foetal bovine serum (FBS) and 1% (*v*/*v*) penicillin-streptomycin (Fisher Scientific, UK)] at 37 °C with 5% CO_2_. The cell confluency was assessed using an Evos digital cell imager (Thermo Fisher, Loughborough, UK) and passaged when they reached 65% confluency. Cells up to passage 20 were used in all assays. Cells were dissociated with TrypLE (Thermo Fisher UK) and centrifuged at 300× *g* for 8 min, followed by cell counting.

### 5.4. MTS Assay

C2C12 cells were seeded into 96-well plates at 4500 cells per cm^2^ in growth media and incubated for 24 h at 37 °C and 5% CO_2_. The required *B. arietans* venom was diluted in growth media, after which it was introduced to C2C12 cells for a further 24 h. At this time, the morphology of the treated cells was imaged at ×10 magnification on an Evos digital imager. Thereafter, the media were replaced with fresh media before incubating with 20 µL of MTS solution (317 µg/mL) (Promega, UK) for 4 h. Spectrophotometric absorbance was measured at 490 nm (FLUOstar OPTIMA, BMG Labtech, Germany).

### 5.5. Scratch Assay

C2C12 cells (40,000 per well) were cultured in 24-well plates until reaching approximately 80% confluency. A scratch was introduced with a p1000 pipette tip down the middle of each well, washed with PBS, and then venom at 0.3125 μg/mL concentrations was diluted in new growth media, which was added to the cells. They were incubated for 24 h under time-lapse microscopy. Images of the cells at the scratch were captured using time-lapse microscopy at 10 min intervals over 24 h (Nikon TiE, Nikon Europe, Amsterdam, The Netherlands). The time point at which the first cell from one side contacted a cell from the other side was taken as wound closure or the migration time.

### 5.6. Cell Migration

C2C12 cells (1500 per well) were seeded into 24-well plates in growth medium and incubated with the desired concentrations of venom (0.3125, 0.157, and 0.078 μg/mL) for 24 h at 37 °C and 5% CO_2_. The images were taken at 10 min intervals for the 24 h period using time-lapse microscopy (Nikon TiE, Nikon Europe, The Netherlands) at ×10 magnification. Migration speed was calculated using the MtrackJ software on ImageJ version 1.54p [51].

### 5.7. Fusion and Atrophy Assays

C2C12 myoblast cells were seeded at 45,000 cells in 1 mL of growth medium on 16 mm acid-treated coverslips (borosilicate glass coverslips, sterilised for 15 min in ≥99% ethanol, followed by hydrochloric acid at 2.5 M, 30 min per side followed by DDH_2_O washes and UV sterilisation) in 12-well plates and incubated at 37 °C and 5% CO_2_ until reaching 80% confluency (~48 h). The growth media was changed to differentiation media containing various concentrations of venom, with a group receiving no venom as a negative control. The cultures were incubated at 37 °C with 5% CO_2_ for 4–5 days. Myotubes were fixed for 15–30 min in 2% (*w*/*v*) paraformaldehyde in PBS 1:1 in the differentiation media after the treatment for both atrophy and fusion. Cells were then washed three times for 5 min with PBS before being stored in the same solution at 4 °C. Immunocytochemistry was performed as described in [23] and below. The fusion index was quantified using Image J and analysed by the ratio of the number of DAPI-stained nuclei inside the myotubes divided by the total number of nuclei per analysed image, and multiplied by 100% to derive the fusion index percentage [51].

A modification of the above was used for the atrophy assay. Cells seeded on treated coverslips in growth media were allowed to reach 85–90% confluency. Growth medium was replaced by differentiation media and cells indued to form of myotubes (4 days). Thereafter, venom in differentiation media was applied for 24 h. Cells were then fixed, and after immunocytochemistry, the myotube area was determined using Image J in myotube that contained more than three or more nuclei [51]. The length and width of the myotubes were used to determine the myotubes area using ImageJ. Multiple measures at different regions of each myotube were taken for width, which were then averaged and multiplied by the length of the myotubes to determine the area [24,51].

### 5.8. In Vivo Venom-Induced Muscle Damage

All animal experiments were conducted following the regulations and principles of the British Home Office for Animals (Scientific Procedures) Act 1986. All procedures used in this study were reviewed and approved by the University of Reading Animal Welfare and Ethics Review Board and the British Home Office (Licence number PP8746932, awarded 6 September 2022). Twenty male C57BL/6 mice (12–13 weeks old, Charles River, Harlow, UK) weighing 25–28 g were allowed to acclimatise for 2 days under standard conditions (12 h light/12 h dark cycle, ad libitum access to food and water). They were then anaesthetised with 3.5% (*v*/*v*) isoflurane in O_2_ and maintained at 2% (*v*/*v*) isoflurane during injections. A quantity of 0.74 µg/g *B. arietans* venom dissolved in 30 µL of PBS total volume was injected intramuscularly into the left Tibialis anterior (TA) muscle of each mouse. The right TA muscle served as a contralateral control. The daily body weights of the mice were taken, and five mice were euthanized at three different time points (day 5, 10, and 20) post injection. The TA muscles of both legs were collected, weighed, and flash frozen over frozen isopentane in a liquid nitrogen beaker. The frozen muscles were transferred into pre-cooled Eppendorf tubes on dry ice and stored at −80 °C. Frozen TA muscles were blocked in OCT (Optimal Cutting Temperature) compound (CellPath Ltd., Newtown, UK). Transverse 15 µm sections were collected using a cryostat (Bright Instruments, Huntingdon, UK) and then transferred onto microscope slides in serial sections before storage at −80 °C.

### 5.9. Histological Staining

Haematoxylin and Eosin (H&E) staining was performed on cryosections as described in other studies [23,51]. Briefly, slides were defrosted and dried for 15 min at room temperature and washed twice in a jar with PBS for two min to remove the OCT. Harris Haematoxylin (Sigma Aldrich, UK) was used for 2 min to stain nuclei and was washed in double-distilled water for same time. The stains were differentiated with two rapid immersions and egressions in solution made of 200 mL 70% ethanol with 200 µL 37% HCl, followed by washing in running tap water for 5 min prior to transferring into 1% Eosin counterstain for 2 min (Sigma Aldrich, UK). Samples were dehydrated through an ethanol series (1 min in 70%, 2 min in 90%, and three washes in 100% ethanol for 2 min each). Slides were placed into xylene for 5 min. Finally, each sample was covered with a glass coverslip fixed using a DPX mounting medium (Fisher, REF D/5319/05). Images were taken with a bright-field microscope (BZ-X810, Keyence Corporation, Itasca, IL, USA).

Picrosirius red staining was performed after warming samples to room temperature for 15 min, followed by immersion in Bouin’s solution [5% (*v*/*v*) acetic acid, 9% (*v*/*v*) formaldehyde, and 0.9% (*v*/*v*) Picric acid] for 15 min in a 56 °C water bath. Samples were then washed under running tap water for 15 min before dipping in Picrosirius red stain (Abcam, UK) for 1 h at room temperature (in the dark). Slides were then briefly dipped twice in 0.5 (*v*/*v*) glacial acidic acid followed by five washes in 100% ethanol, and once for 5 min in xylene. Finally, DPX mounting medium was used to fix the coverslips on the sample. Imaging was performed using bright-field microscopy (BZ-X810, Keyence Cooperation, Itasca, IL, USA).

### 5.10. Immunohistochemistry (IHC)

IHC was performed on cells and muscle sections as described in Richards et al. [51] and Williams et al. [23]. Briefly, slides were removed from the −80 °C freezer, warmed, and dried for 15 min at room temperature. A hydrophobic barrier was drawn around the desired sections using a PAP pen and left to dry for 15 min to ensure proper binding with antibodies. For IHC of the cells, preparations were allowed to reach room temperature. The samples were washed three times in PBS for 5 min before incubating in permeabilization buffer [20 mM HEPES pH 7, 300 mM sucrose, 50 mM NaCl, 3 mM MgCl_2_, and 0.5% (*v*/*v*) Triton X-100] for 15 min. Thereafter, samples were washed three times with PBS, followed by incubation in blocking-wash buffer, [5% (*v*/*v*) horse serum, 0.05% (*v*/*v*) Triton X-100, and 25 mg Sodium Azide in PBS] for 30 min. Primary antibodies were pre-incubated in a blocking-wash buffer for 30 min before being added to samples at 4 °C overnight. This was followed by three washes with a blocking-wash buffer for 10 min at room temperature before incubating for 1 h in the dark with secondary antibodies conjugated with different Alexa Fluor™ fluorophores (blocked using blocking-wash buffer for a minimum of 30 min before use). Subsequently, samples were washed with blocking-wash buffer as before, then coverslips were mounted under a drop of DAPI (1:2000 DAPI with Dako fluorescence mounting medium). The slides were imaged with fluorescence microscopy (BZ-X810, Keyence Cooperation, USA) at ×10 magnification and analysed with ImageJ.

The primary antibodies used in this study were Mouse anti- Pan Myosin heavy chain (Clone A14.1025 DSHB, USA cell supernatant) 1:200, Mouse anti-Myosin Heavy Chain 3, 1:200, (Santa Cruz, SC53091, USA), Rabbit anti-Laminin, 1:200, (Sigma Aldrich, L9393, UK), Rabbit anti-Dystrophin, 1:200, (Abcam 15,277), Rabbit anti-Desmin, 1:500 (Ab 15200), Rat Alexa Fluor 647 conjugated anti-P-selectin, 1:500 (BD Biosciences (Franklin Lakes, NJ, USA) RB40.34), Rabbit FITC conjugated anti Fibrinogen 1:500 (DAKO F0111). The secondary antibodies were Goat anti-Mouse or anti-Rabbit IgG, 1:200, either Alexa Fluor 488 or 594 (A11029, A11032, A11034, A11037, Invitrogen, Paisley, UK).

For quantification, the following fields of view areas were used: 800 × 800 µm for H&E and Picrosirius red, and 200 × 200 µm for IgG, Myosin heavy chain three (MYHC3, embryonic myosin), Laminin, and Dystrophin. IgG infiltration area, MYHC3, and Laminin intensity were measured using ImageJ. For Laminin, lines were drawn across numerous points of the thickness of the laminin surrounding each of the fibres within the 200 × 200 µm selected areas. The maximum grey value intensity of the lines for each field of view was averaged.

The dystrophin perimeter circularity was measured for each fibre within 200 × 200 µm fields of view. Expression around the full perimeter was set at 100% and fibres not expressing around the entirety of perimeter presented as a % of the total.

### 5.11. Statistical Analysis

Statistical analyses were performed using GraphPad Prism 10 (Dotmatics, Boston, MA, USA). To test the effect of a single factor with more than two levels, between independent samples (for example multiple concentrations of a treatment), one-way Analysis of Variance (ANOVA) was used to test the overall effect of the factor (for example: *Treatment*). The normality of the data distribution was assumed but not tested, due to the robustness of the one-way ANOVA as a hypothesis testing tool, even in violation of data normality (10.7334/psicothema2016.383). Pairwise comparisons between control and different levels of treatment were performed using unpaired *t*-tests with Bonferroni correction for multiple comparisons. To test the effect of a factor *between* two independent groups, we performed unpaired Student’s *t*-tests and reported the *p* value. Data are shown as the mean ± standard error of the mean (SEM).

## Data Availability

The original contributions presented in this study are included in the article. Further inquiries can be directed to the corresponding authors.
